# A J-Like Protein Influences Fatty Acid Composition of Chloroplast Lipids in Arabidopsis

**DOI:** 10.1371/journal.pone.0025368

**Published:** 2011-10-18

**Authors:** Imad Ajjawi, Ardian Coku, John E. Froehlich, Yue Yang, Katherine W. Osteryoung, Christoph Benning, Robert L. Last

**Affiliations:** 1 Department of Biochemistry and Molecular Biology, Michigan State University, East Lansing, Michigan, United States of America; 2 Michigan State University (MSU)–Department of Engineering (DOE) Plant Research Laboratories, Michigan State University, East Lansing, Michigan, United States of America; 3 Department of Plant Biology, Michigan State University, East Lansing, Michigan, United States of America; United States Department of Agriculture, Agricultural Research Service, United States of America

## Abstract

A comprehensive understanding of the lipid and fatty acid metabolic machinery is needed for optimizing production of oils and fatty acids for fuel, industrial feedstocks and nutritional improvement in plants. T-DNA mutants in the poorly annotated *Arabidopsis thaliana* gene At1g08640 were identified as containing moderately high levels (50–100%) of 16∶1Δ7 and 18∶1Δ9 leaf fatty acids and subtle decreases (5–30%) of 16∶3 and 18∶3 (http://www.plastid.msu.edu/). TLC separation of fatty acids in the leaf polar lipids revealed that the chloroplastic galactolipids monogalactosyldiacylglycerol (MGDG) and digalactosyldiacylglycerol (DGDG) were the main lipid types affected by this mutation. Analysis of the inferred amino acid sequence of At1g08640 predicted the presence of a transit peptide, three transmembrane domains and an N-terminal J-like domain, and the gene was named *CJD1* for *Chloroplast J-like Domain 1*. GFP reporter experiments and *in vitro* chloroplast import assays demonstrated CJD1 is a chloroplast membrane protein. Screening of an Arabidopsis cDNA library by yeast-2-hybrid (Y2H) using the J-like domain of CJD1 as bait identified a plastidial inner envelope protein (Accumulation and Replication of Chloroplasts 6, ARC6) as the primary interacting partner in the Y2H assay. ARC6 plays a central role in chloroplast division and binds CJD1 via its own J-like domain along with an adjacent conserved region whose function is not fully known. These results provide a starting point for future investigations of how mutations in CJD1 affect lipid composition.

## Introduction

As the site of photosynthesis the chloroplast is the defining organelle of plant cells. In addition to its role in biomass accumulation through carbon fixation, it participates in a wide range of biosynthetic processes ranging from production of the hormone jasmonate to synthesis of nutritionally important vitamins, amino acids and lipids. Proteomics and DNA sequence analysis indicate that the chloroplast contains several thousand proteins [1]–[3], and the vast majority are encoded by nuclear genes. Despite decades of research on chloroplast biology, the function of a relatively small fraction of these proteins is well defined.

The Chloroplast 2010 project (http://www.plastid.msu.edu/) is a large-scale reverse genetics mutant screen that aims at improving the annotation of nuclear genes encoding chloroplast targeted proteins. Approximately 5,500 *Arabidopsis thaliana* T-DNA lines with homozygous mutations in 3,400 nuclear genes predicted to encode plastid-targeted proteins have been analyzed thus far. Major goals of this project are to associate phenotypes with these mutant lines [4], and to identify pleiotropic syndromes due to unexpected connections between plastidial processes [5]. To achieve these objectives, the T-DNA lines were subjected to a battery of phenotypic assays that capture morphological, chemical and physiological traits [5]. The results collected by the Chloroplast 2010 Project pipeline are stored in a relational database and are freely available for query at http://bioinfo.bch.msu.edu/2010_LIMS [6].

A complete understanding of the plant lipid metabolic machinery is essential for rational engineering of oils and fatty acids for fuel, industrial feedstocks and nutritional improvement [7]–[9]. Extensive Arabidopsis forward genetic mutant screens for changes in leaf fatty acids by analysis of fatty acid methyl esters (FAMEs) [10] played a prominent early role in establishing key steps in fatty acid desaturation and acyl-lipid metabolism [10] [11]–[16]. Since then several hundred Arabidopsis genes were identified or hypothesized to play a role in lipid metabolism based upon experimental evidence or genomics (for a recent comprehensive overview of genes involved in *A. thaliana* acyl-lipid metabolism see [17]). Despite the large body of work pre-dating the Chloroplast 2010 Project, novel fatty acid mutants were identified in the project pipeline; for published examples see [4]. In some cases the mutations affected genes with known roles in acyl-lipid metabolism yet helped refine the current understanding of these processes. For instance, two mutants with abnormal fatty acid composition were identified for acyl carrier protein 4 (ACP4; [4]), a cofactor that plays a key role in fatty acid biosynthesis [18]–[20]. Another mutation identified in the pipeline linked a gene with reportedly unrelated function to fatty acid metabolism. In this case mutants and lines overexpressing the Arabidopsis gene At1g10310, annotated as a pterin aldehyde reductase [21], were shown to contain abnormal levels of 18 carbon seed fatty acids [4].

Here we describe mutants of At1g08640, a poorly annotated gene with defects in the fatty acid composition of chloroplast-specific galactolipids. The gene encodes a chloroplast membrane protein with a DnaJ-like domain. Our results are consistent with the hypothesis that the protein resides in the inner envelope membrane and is capable of interacting with the ARC6 protein, a key component of the chloroplast division pathway [22], [23].

## Results

### Mutation of unnannotated gene At1g08640 results in abnormal fatty acid profiles

**Figure 1:**
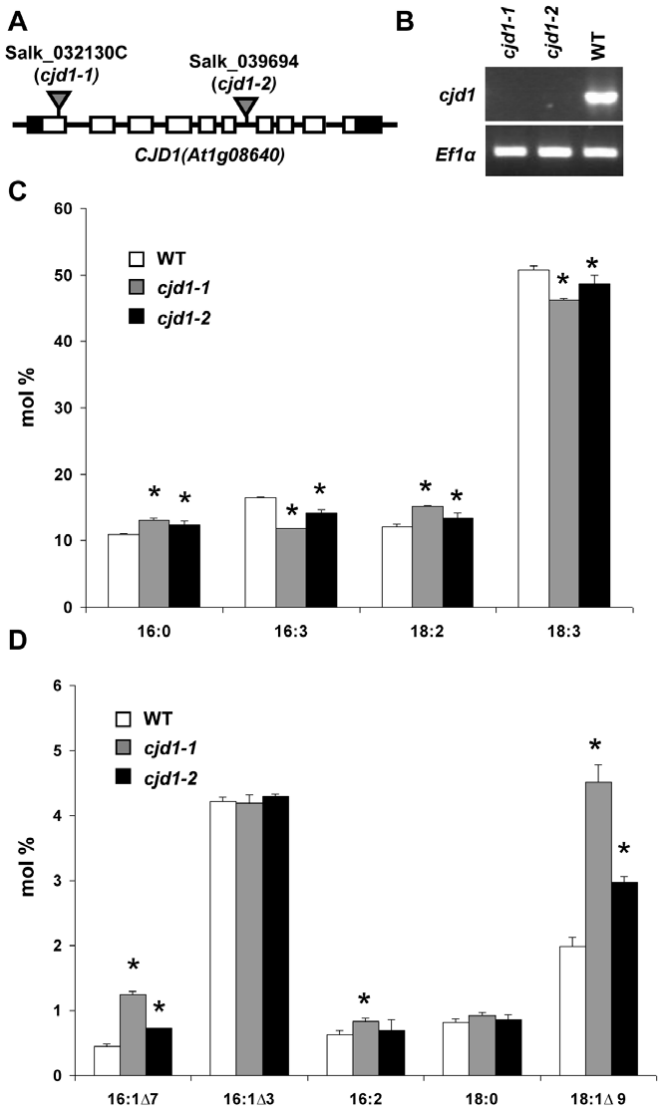
T-DNA mutants in *CJD1* possess altered fatty acid profiles. A, Salk_032130C (*cjd1-1*) contains a T-DNA insertion in the first exon of *CJD1* (At1g08640) while Salk_039694 (*cjd1-2*) harbors a T-DNA insertion in intron 6. T-DNA insertions are illustrated as triangles; exons, introns and untranslated regions are depicted by empty rectangles, solid lines and black rectangles, respectively. B, Semi-quantitative RT-PCR analysis shows that leaves of *cjd1-1* and *cjd1-2* do not accumulate detectable *CJD1* transcript under the conditions tested. Wild-type plants (WT) and the elongation initiation factor 1 alpha (EFlα, GenBank accession no. X16432) were used as controls. C, and D, FAME profiles from GC-FID expressed in mol % for WT, *cjd1-1* and *cjd1-2*. The error bars represent the standard deviation of three biological replicates and statistically significant differences relative to wild type (Student's *t* test *P*<0.01) are indicated with asterisks.

*A. thaliana* T-DNA mutant Salk_032130C, which contains an insertion in exon 1 of At1g08640, named *CJD1* for ***C*** *hloroplast* ***J*** *-like* ***D*** *omain 1*, was found to have an unusual FAME profile in the Chloroplast 2010 Project pipeline (http://bioinfo.bch.msu.edu/2010_LIMS). The most striking change was a moderate increase (50–100%) in monounsaturated fatty acids 16∶1Δ^7^ (16∶1, number of carbons∶number of double bonds; Δ^7^, double bond between carbon 7 and 8 counting from the carboxyl end) and 18∶1Δ^9^ (Fig. 1d). These changes were accompanied by more subtle decreases (5–30%) of the corresponding 16∶3 and 18∶3 polyunsaturated fatty acids (Fig. 1c) along with smaller but statistically significant increases (Student's *t* test *P*<0.01) in 16∶0, 16∶2 and 18∶2. We confirmed that the mutation in *CJD1* was responsible for the syndrome of phenotypes by showing that homozygous individuals bearing a second mutant allele, Salk_039694 (Fig. 1a), possessed alterations in fatty acid profile similar to those observed for Salk_032130C (Fig. 1c,d). CJD1 RNA was undetectable by RT-PCR in plants homozygous for either mutant allele (Fig. 1b), confirming that the observed fatty acid changes were caused by decreased *CJD1* expression.

### DNA sequence analysis predicts that *CJD1* encodes a plastidic intrinsic membrane protein with a J-like domain

**Figure 2:**
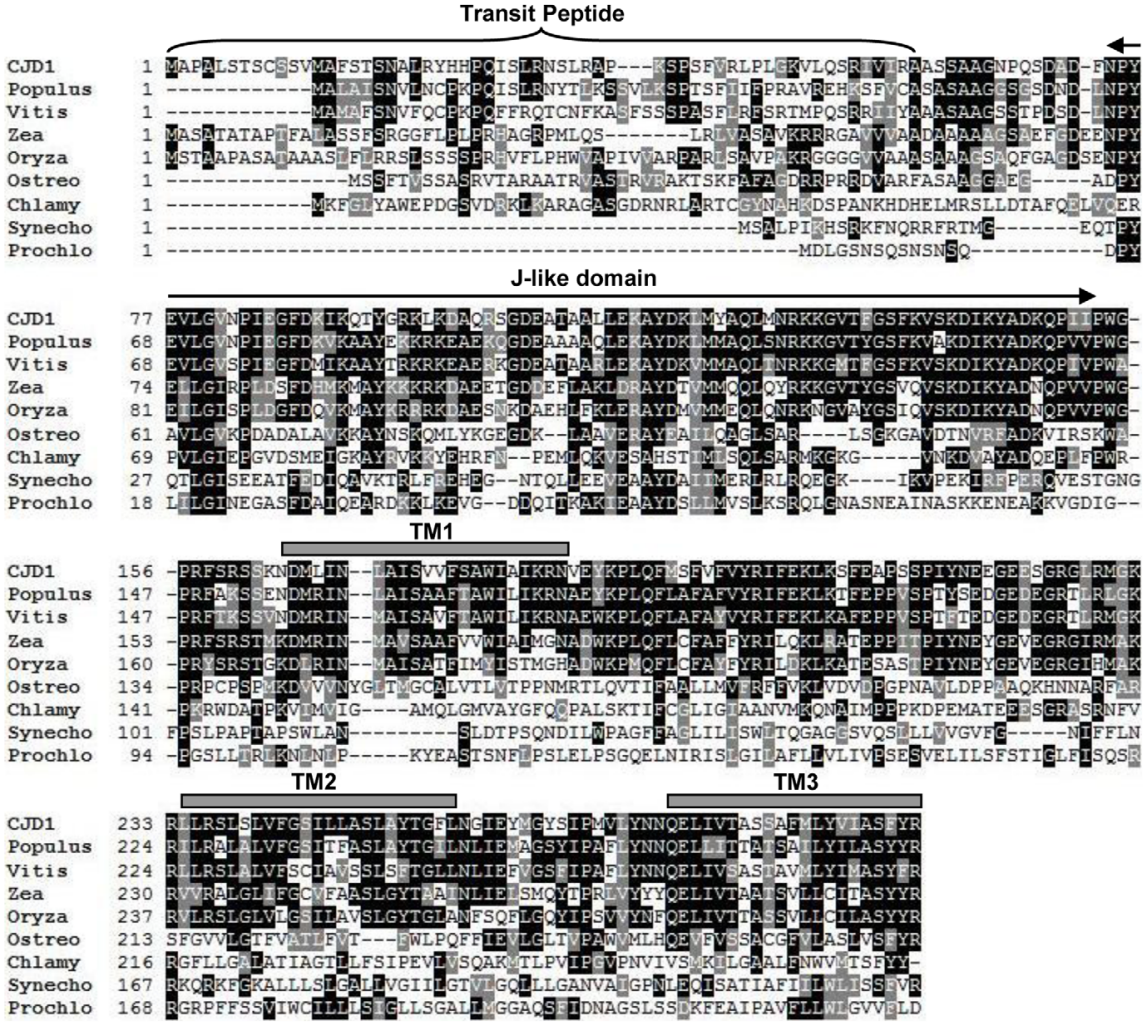
Analysis of Arabidopsis CJD1 inferred amino acid sequence. Clustal W (1.83) alignment of CJD1 with selected homologues. Identical residues are depicted by black boxes while similar residues are shaded with grey boxes. The bracket delineates the predicted transit peptide, grey bars indicate predicted transmembrane domains (TM1, 2 and 3) and the double arrow defines the J-like domain. Abbreviations and GenBank Protein ID: CJD1, 18390922, Populus, *Populus trichocarpa*, 222862208; Vitis, *Vitis vinifera*, 225453038; Zea, *Zea mays*, 194705880; Oryza, *Oryza sativa* (japonica cultivar), 78708817, Ostreo, *Ostreococcus lucimarinus*, 145347386; Chlamy, *Chlamydomonas reinhardtii*, 159491044; Synecho, *Synechocystis* sp. (PCC6803), 16329734; Prochlo, *Prochlorococcus marinus* (NATL2A), 72001786.

*In silico* analysis of the At1g08640 predicted protein sequence reveals an evolutionarily conserved protein with three major features (Fig. 2). First, the N-terminal 60 amino acids contain features of a canonical chloroplast transit peptide (TargetP) [24], consistent with a plastid localization. Second, the N-terminal 74 to 153 amino acids of the predicted mature protein bear a resemblance to known J-like domains [25] (and see below). Finally, transmembrane domain prediction algorithms [26] identified three putative transmembrane domains distributed throughout the rest of the protein (Fig. 2). This analysis suggests that *CJD1* encodes a plastidic intrinsic membrane protein. A BLAST search [27] for CJD1 homologues revealed that the whole protein, including the three predicted transmembrane domains, is well-conserved among plants (Fig. 2) and is specific to photosynthetic organisms. Phylogenetic analysis of CJD1-related proteins indicates that algal sequences have weaker similarity to land plant CJD1. In addition, cyanobacterial homologues are even more distantly related and form a clade distinct from the eukaryotic proteins (Fig. S1). Despite its widespread occurrence no functional annotation was found for CJD1 protein or its homologues in The Arabidopsis Information Resource annotation version 9 or in GenBank.

### CJD1 is a chloroplast membrane protein

**Figure 3:**
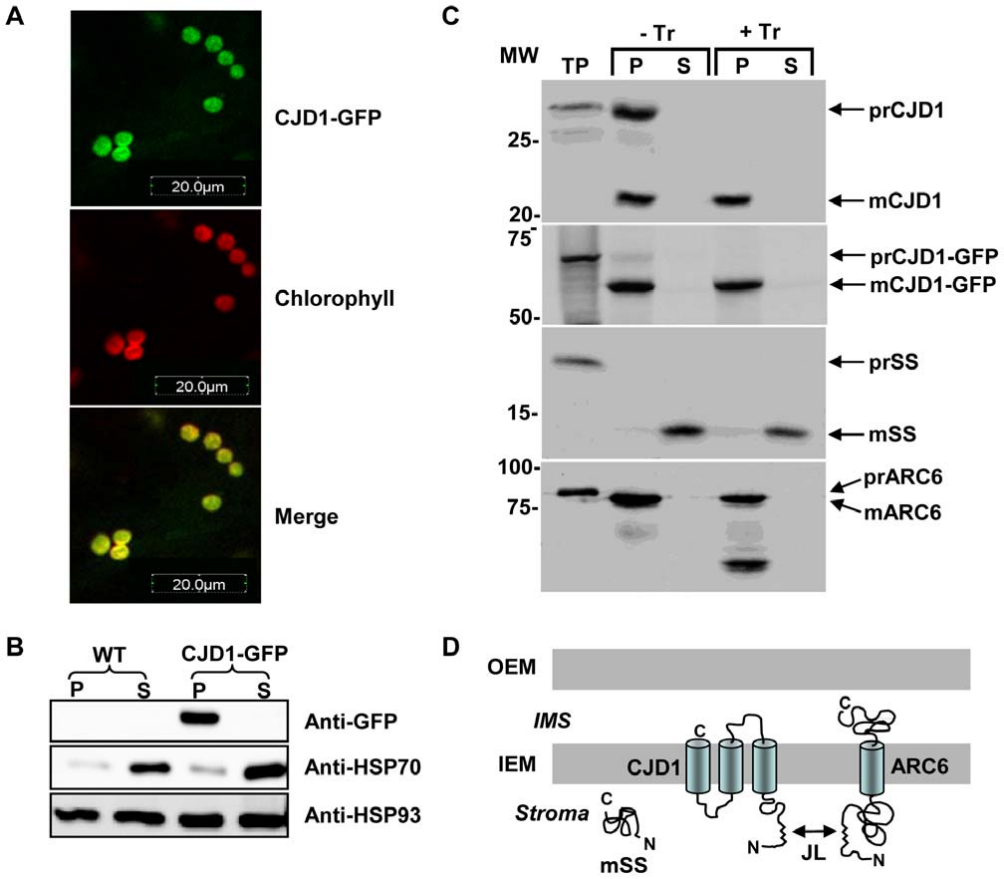
CJD1 protein resides in chloroplast membranes. A, Confocal images of Arabidopsis leaves expressing CJD1-GFP indicate that the fusion protein is targeted to chloroplasts. B, Immunoblotting of fractionated chloroplasts (membrane, P; soluble, S) probed with anti-GFP, anti-HSP70 and anti-HSP93. WT, untransformed wild-type plants; CJD1-GFP, transgenic lines expressing CJD1-GFP. C, Chloroplast import experiments with radiolabeled recombinant CJD1, CJD1-GFP, ARC6 and rubisco small subunit (SS). Chloroplasts were isolated following treatment with (+Tr) or without Trypsin (−Tr) and fractionated into membrane (p) and soluble fractions (s). TP, translation product; MW, molecular weight; m, mature protein; pr, precursor protein. D, Predicted CJD1 topology based on import assay results, published proteomics studies [28]–[30] and the location of putative transmembrane domains. OEM, chloroplast outer membrane; IEM, chloroplast inner membrane; IMS, chloroplast intermembrane space; Stroma, chloroplast stroma; JL, J-like domain.

The informatically predicted chloroplast membrane localization of CJD1 protein is consistent with published proteomics evidence that CJD1 is found in chloroplast envelope preparations [28]–[30], and was tested experimentally by complementary methods. First, the C-terminus of the complete CJD1 open reading frame was fused to the N-terminus of the green fluorescent protein (GFP) and stably transformed into Arabidopsis using *Agrobacterium tumefaciens*. GFP fluorescence was confined to chloroplasts as observed in confocal images taken from transgenic leaves (Fig. 3a). To test the bioinformatic prediction that CJD1-GFP would be in chloroplast membranes, intact chloroplasts were isolated, lysed, fractionated and the resulting soluble and membrane fractions were assayed by immunoblot analysis with anti-GFP antibody following SDS-PAGE. In contrast to soluble HSP70 protein [31], [32], which was enriched in soluble stromal fractions of untransformed and transformed lines, CJD1-GFP was detected only in the membrane fraction of transgenic lines (Fig. 3b). HSP93 was present ubiquitously in all fractions as previously reported [33], [34]. Taken together these data are consistent with the hypothesis that CJD1 is a chloroplast membrane protein.

To explore whether the protein is present in the outer or inner envelope membrane and analyze the topology of the protein within the membrane, native CJD1 and CJD1-GFP preproteins were subjected to chloroplast import and protease sensitivity assays. Consistent with chloroplast localization of CJD1-GFP fluorescence, radiolabeled proteins were imported and processed by purified pea chloroplasts (Fig. 3c). An ∼7 KDa shift was observed between the full-length precursor protein translation products and the mature proteins, confirming the presence and approximate size of the predicted transit peptide (Fig. 3c; top and second panels). As expected [22], [23], the positive control inner envelope single membrane spanning domain protein ARC6 was sensitive to Trypsin treatment, whereas the stromal small subunit of Rubisco protein was insensitive to protease digestion. The radiolabeled CJD1 protein was protected from Trypsin digestion even when fused to GFP, suggesting that CJD1 is not located in the outer envelope nor accessible to protease in the intermembrane space. This is consistent with published proteomics data from three different studies indicating that CJD1 is found in chloroplast envelope preparations [28]–[30]. Taking topological considerations into account [35], we hypothesize that the N-terminal portion of mature CJD1 protein faces the stroma (Fig. 3d). This hypothetical topology model is consistent with the observed interaction of the CJD1 J-like domain with the inner envelope protein ARC6 in the yeast 2-hybrid (Y2H) system (see below).

### The fatty acid phenotype is specific to chloroplast galactolipids MGDG and DGDG

Based upon the chloroplast localization of CJD1 protein we hypothesized that the fatty acid composition of chloroplast lipids would be most severely affected in *cjd1* plants. To test this idea, the most abundant leaf polar lipids (phosphatidylcholine (PC), phosphatidylethanolamine (PE), phosphatidylglycerol (PG), monogalactosyldiacylglycerol (MGDG) and digalactosyldiacylglycerol (DGDG)) were separated on silica plates by thin layer chromatography and the fatty acids in individual lipid classes were analyzed by transesterification to produce FAMEs [36]. This secondary assay revealed that the fatty acid composition of the plastidic galactolipids MGDG and DGDG were the most noticeably altered, though less consistent changes were also observed for PG and PC (Table 1). In MGDG and DGDG, increases ranging from 0.5 to 3 fold were observed for 16∶0, 16∶1Δ^7^ and 18∶1Δ^9^ (Table 1), while decreases ranging from 15 to 50% were observed for DGDG-specific 16∶3 and 18∶3. An ∼20% decrease was also observed for 16∶3 in MGDG, but not for 18∶3. No changes in the amount of each lipid class were noted. Together, these data indicate that polyunsaturated fatty acids of chloroplastic galactolipids are reduced, while monounsaturated fatty acids and, to a lesser extent, saturated fatty acids, are increased. FAD mRNA levels were analyzed to test the hypothesis that the *cjd1* mutations' influence on polyunsaturated fatty acids was due to changes in expression of fatty acid desaturase gene expression. However, RT-PCR experiments revealed no differences in mRNA accumulation for the chloroplast fatty acid desaturases *FAD5*, *6* and *7* between wild type and *cjd1* mutants (Fig. S2).

**Table 1:** Fatty acid composition of leaf glycerolipids of wild-type and cjd1 mutant plants.

Lipid	16∶0	16∶1Δ7	16∶1Δ3	16∶2	16∶3	18∶0	18∶1Δ9	18∶2	18∶3
**Monogalactosyldiacylgycerol**									
WT	1.2±0.1	1.1±0.1	1.8±0.2		34.8±0.3	0.2±0.07	1.0±0.1	2.5±0.1	57.2±0.4
*cjd1-1*	3.1±0.3***	3.0±0.1***	1.9±0.08		26.8±0.5***	0.3±0.2	3.3±0.2***	3.4±0.3**	58.0±0.7
*cjd1-2*	2.2±0.05***	2.3±0.1***	1.8±0.05		29.7±0.3***	0.2±0.07	2.2±0.09***	3.0±0.08**	58.6±0.5*
**Digalactosyldiacylglycerol**									
WT	16.5±1.8			0.7±0.06	3.2±0.2	1.2±0.2	1.4±0.2	4.5±0.2	72.3±2.2
*cjd1-1*	26.4±0.7***			0.4±0.02***	1.6±0.05***	1.7±0.05*	4.2±0.3***	6.7±0.3***	58.8±1.3***
*cjd1-2*	24.8±0.5***			0.4±0.01**	1.9±0.03***	1.7±0.04*	2.8±0.2***	6.1±0.1***	61.9±0.8***
**Phosphatidylglycerol**									
WT	26.2±1.0		37.4±1.7			1.1±0.2	5.3±0.6	6.5±0.5	23.4±1.0
*cjd1-1*	25.9±0.5		37.7±0.6			1.3±0.1	11.5±0.5***	7.3±0.4	16.4±0.8***
*cjd1-2*	26.9±1.6		41.9±1.4*			8.5±0.5†	7.0±0.4	20.0±1.5*	
**Phosphatidylethanolamine**									
WT	35.3±0.2					2.8±0.09	2.8±0.3	38.8±1.1	20.2±1.0
*cjd1-1*	35.8±0.5					2.8±0.3	3.5±0.2*	39.3±0.5	18.6±0.4*
*cjd1-2*	36.5±0.9					2.7±0.2	3.3±0.2	37.7±1.3	19.8±1.7
**Phosphatidylcholine**									
WT	24.2±1.7					2.9±0.01	8.8±0.6	36.2±1.3	28.0±1.2
*cjd1-1*	24.5±1.0					2.9±0.2	11.7±0.6***	37.2±0.5	23.7±0.5***
*cjd1-2*	29.1±6.2					3.7±1.0	11.7±0.6***	34.2±3.5	21.2±4.0*

**Values shown are mol % and means of n = 4 for WT and n = 3 for** ***cjd1-1*** **and** ***cjd1-2*** (where n is a biological replicate). Statistically significant values relative to WTare indicated (Student's t test, * ***P*** <0.05, ** ***P*** <0.01, *** ***P*** **<0.001). Fatty acid values that could not be determined (due to the limit of detection or because they are not present in that lipid species) were left blank.**

† **n = 1.**

### Analysis of the J-like domain

**Figure 4:**
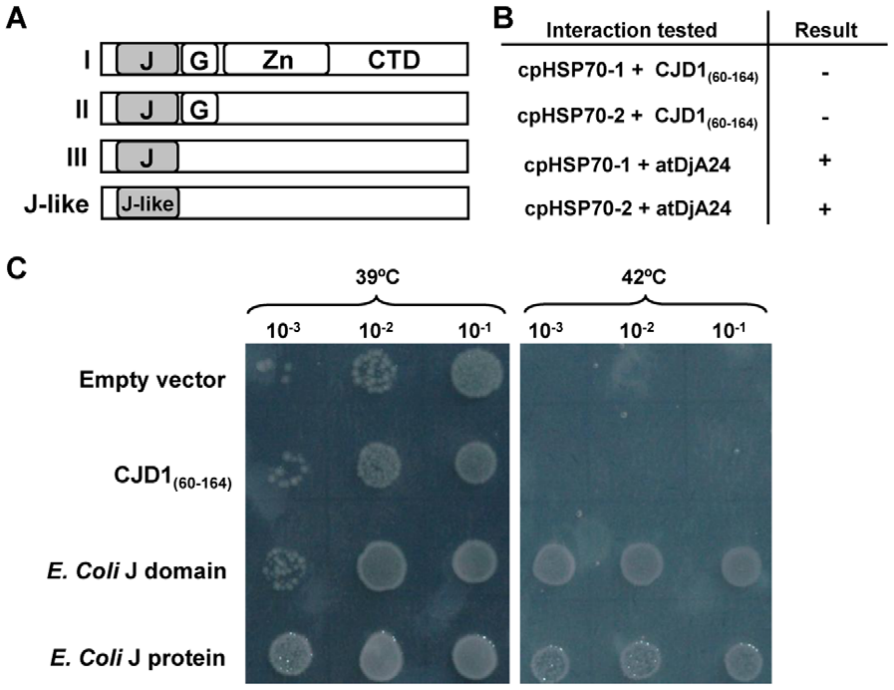
Assay of CJD1 J-like domain as a possible co-chaperone. A, Modular organization and classification of the different types of J proteins (I, II, III and J-like) proposed by [25]. J, J domain; G, Glycine rich domain; Zn, Zn-finger domain; CTD, C-terminal domain. B, Results of Y2H experiments with the two Arabidopsis chloroplastic HSP70 proteins and CJD1 J-like domain or atDjA24 HSP40 co-chaperone J-domain. C, The J-like domain of CJD1 does not rescue the temperature sensitivity of an *E. coli dnaJ*/*cbpA* double knockout mutant. The empty vector and CJD1_60–164_ transformed mutants were viable at 39°C, but inviable at 42°C, while the cells transformed with the full *E. coli* DnaJ protein and only the J domain were viable at both temperatures. Cells were spotted on LB media supplemented with 0.5% w/v arabinose and 20 µg/ml ampicillin.

Secondary structure profiling [37] and hidden Markov models [38] predict an 80 amino acid domain at the N-terminus of the mature CJD1 protein that resembles J domains of DnaJ proteins (Fig. S3). Typical DnaJ proteins, also known as HSP40s, are co-chaperones defined by a J domain that can bind DnaK/HSP70 protein and stimulate its ATPase activity [39]. As diagrammed in Figure 4a, there are 3 types of documented J proteins. While canonical HSP40 co-chaperones fall into type I and contain an N-terminal J domain, followed by a glycine-rich region, a Zn-finger domain and a C-terminal domain, their structures vary. J domains of the sort found in HSP40 and other chaperones are approximately 70 amino acids in length and consist of 3 to 5 helices with a conserved HPD amino acid triptych between helices II and III [25]. J domains lacking the HPD motif are classified as J-like domains; CJD1 falls into this category since it only contains a J-like domain that lacks the HPD triptych between predicted helices. To determine whether CJD1 is capable of acting as a co-chaperone we tested whether the CJD1 J-like domain could functionally replace *E. coli* HSP40 *in vivo*. The *E. coli dnaJ/cbpA* double knockout mutant is incapable of growth at 42°C or higher, but can be rescued by transformation with functional J domains or the full *E. coli* HSP40 protein [40], [41]. Expression of the full-length *E. coli* DnaJ protein using the *E. coli* P_BAD_ arabinose inducible promoter reversed the temperature sensitivity, confirming that the assay worked in our hands (Fig. 4c). Expression of the *E. coli* DnaJ domain alone (amino acids 1–108 of HSP40) also complemented the temperature sensitivity, indicating that the J domain alone could act as a co-chaperone in this assay. In contrast, the analogous Arabidopsis J-like domain fragment, CJD1_60–164_, failed to rescue the temperature sensitivity of the *dnaJ*/*cbpA* double knockout (Fig. 4c) even at high arabinose concentrations.

An alternative approach was used to determine whether the J-like domain of CJD1 is capable of binding HSP70. In this case, the interaction between each of the two Arabidopsis chloroplast stromal HSP70s (At4g24280; cpHSP70-1 and At5g49910; cpHSP70-2) [31] and the J-like domain (CJD1_60–164_), was tested by Y2H assay. Yeast cells carrying either Arabidopsis cpHSP70-1 or cpHSP70-2 and CJD1_60–164_, were incapable of growing on selective medium (Fig. 4b) indicating that CJD1_60–164_ and the two Arabidopsis stromal HSP70 proteins do not bind strongly enough to give a positive Y2H result.

Although this result suggests that the CJD1 J-like domain does not bind HSP70, an alternative hypothesis is that even *bona fide* plant J domain-HSP70 interaction would not be strong or stable enough to yield a positive Y2H result. To address this hypothesis, the J-domain from the chloroplastic type I HSP40 At4g39960 (atDjA24, [42]) was used as a bait with both stromal cpHSP70-1 and -2, yielding a positive result in both cases (Fig. 4b). This result is reminiscent of the reported positive Y2H result obtained between human HSP70 and full-length human HSP40 [43] and strengthens the hypothesis that the CJD1 J-like domain does not form a stable interaction with chloroplastic HSP70. Taken together, the Y2H and *E.coli* temperature sensitive mutant experiments argue that the J-like domain of CJD1 is neither capable of binding HSP70s in yeast nor of stimulating ATPase activity in *E. coli*. These results support the *in silico* observation that the protein does not contain a canonical J domain.

### ARC6 protein interacts with the CJD1 J-like domain in yeast

**Figure 5:**
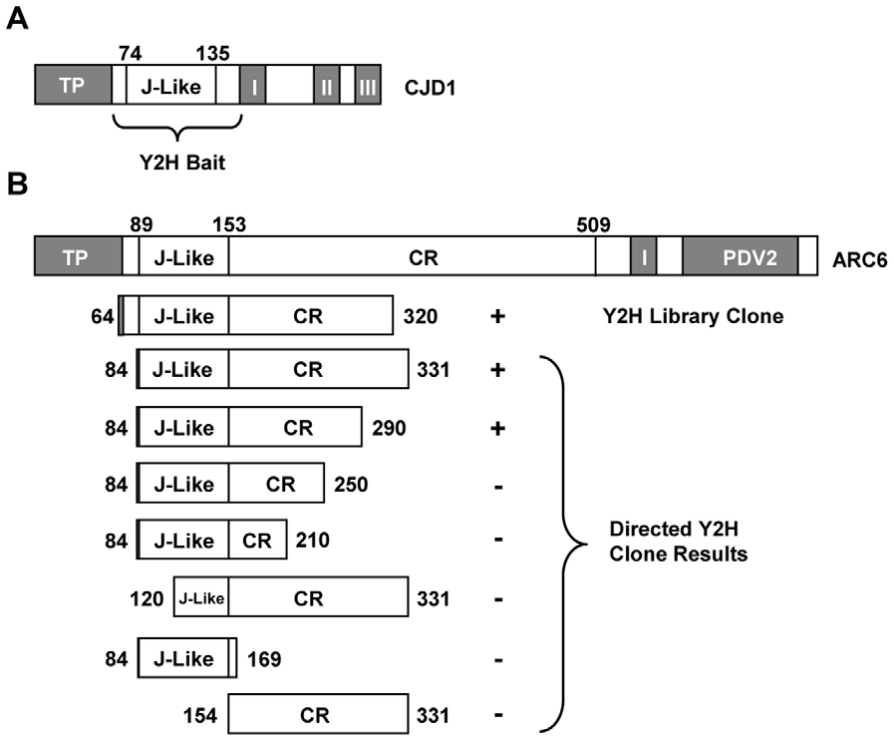
Schematic representation of selected yeast 2-hybrid clones and results. TP, transit peptide; Roman numerals, transmembrane domains; CR, conserved region; PDV2, PDV2 binding domain. A, CJD1 protein. The amino terminal soluble portion (amino acids 60–164) was used as the bait. This peptide includes the J-like domain (amino acids 74–135). B, ARC6 clones and Y2H results. Top drawing: modular organization of ARC6. Second drawing: representative clone identified by library screening. This clone has the full J-like domain and the shortest CR domain of the six clones recovered (see Table 2 for details of other clones). Constructs defined by bracket: results from directed Y2H screening of truncated ARC6 proteins with positive and negative results indicated with ‘+’ and ‘−’, respectively.

The strong resemblance between the predicted 3-D structure of the CJD1 J-like domain (Fig. S3) and J domains of HSP40 chaperones suggested the hypothesis that it binds other proteins in carrying out its biological function. To pursue this idea, a cDNA library from one-week old Arabidopsis seedlings [44] was screened for potential J-like domain interacting proteins by Y2H. A construct containing the J-like domain (CJD1_60–164_) was used as bait for the Y2H screen (Fig. 5a). Sixteen independent clones corresponding to eight different proteins were retrieved during the Y2H screen (Table 2). Out of the eight proteins, two are unlikely to be biologically relevant interactors (At1g05600 and At1g08800) because the prey fragment was either out of frame with the GAL4 activation domain or present in the antisense orientation. Four other protein candidates were not predicted to reside in the chloroplast (At5g60410, At5g53140, At3g53690 and At2g22100). Of the two proteins thought to be in the chloroplast (At2g35500 and At5g42480), At5g42480 (also known as ARC6) was the best candidate because six positive colonies encoding five different peptides were identified for ARC6 (see Table 2 for details) while only one positive colony was observed for At2g35500. The library screening results were confirmed by using CJD1_60–164_ as both bait (as in the original library screen) and prey with a construct containing ARC6_84–331_ (Fig. 5b, third from top).

**Table 2:** Clones retrieved by Yeast 2 hybrid screening.

Locus	Annotation	Start	Stop	Localization
At5g42480	ARC6	129	1020	Chloro IM (Exp)
At5g42480	ARC6	159	1030	Chloro IM (Exp)
At5g42480	ARC6	159	1030	Chloro IM (Exp)
At5g42480	ARC6	174	1265	Chloro IM (Exp)
At5g42480	ARC6	192	961	Chloro IM (Exp)
At5g42480	ARC6	240	968	Chloro IM (Exp)
At1g05600†	Pentatricopeptide repeat (PPR) containing	418	−91	Mito/Chloro (in silico)
At5g60410	ATSIZ1/SIZ1	387	1984	Nucleus (Exp)
At2g22100	RNA recognition motif (RRM) containing	3	540	Nucleus/Mito (in silico)
At1g08800††	Unknown	472	3070	ER (in silico)
At1g08800††	Unknown	472	3070	ER (in silico)
At5g53140	Protein phosphatase 2C, putative	495	978	Unknown
At5g53140	Protein phosphatase 2C, putative	495	978	Unknown
At5g53140	Protein phosphatase 2C, putative	495	978	Unknown
At2g35500	Shikimate kinase-related	15	1090	Chloro (MS)
At3g53690	Zinc finger family protein	−7	687	Unknown

Start/Stop indicate the position of the 5′ and 3′ prey fragment ends, relative to the position of the ATG start codon (A = 0).

† Antisense in respect to the reference sequence.

†† Out of frame with the GAL4 activation domain.

Chloro, chloroplast; IM, inner membrane; Mito, mitochondria; Exp, experimentally determined; MS, detected by Mass Spectrometry; Unknown, no evidence for subcellular localization.

ARC6 is a chloroplast inner envelope membrane protein that is a key player in assembling the chloroplast division complex [22], [45]. The ARC6 clones identified from the Y2H screen all encode stromal ARC6 peptides (Fig. 5b), consistent with the topology model predicting that CJD_60–164_ is in the stroma (Fig. 3d). Interestingly, the N-terminal region of ARC6 also contains a J-like domain. This is followed by a region that is well-conserved in ARC6 homologues (designated “CR” for Conserved Region; Fig. 5b [22], [23]). The function of the CR has not been fully characterized, though this region has been shown to interact with the chloroplast division protein FtsZ2 [46].

The five ARC6 proteins that yielded positive Y2H results included the entire J-like domain and ∼170–270 amino acids of the CR (Table 2). To determine whether the full ARC6 J-like domain and entire 170 amino acids of the CR are both required for binding to CJD1_60–164_, the region was subdivided as shown in Figure 5b. Because ARC6_84–290_ was the only subclone that yielded a positive Y2H result, we conclude that the J-like domain and first ∼140 amino acids of the CR of ARC6 are each necessary but not sufficient for a positive Y2H result with CJD1_60–164_.

### Generation and analysis of a CJD1/ARC6 double mutant

The interaction between the stromal region of ARC6 and CJD1 J-like domain is consistent with the hypothesis that CJD1 protein is present in the inner envelope with the topology shown in Fig. 3d, and suggested that an interaction with ARC6 protein may be important for function of CJD1 or ARC6 or both. Because both are intrinsic membrane proteins, making biochemical analysis problematic, genetic evidence was sought to explore the functional significance of the Y2H results *in vivo*. We hypothesized that, if CJD1-ARC6 interaction is essential for the function of either protein, an *arc6* mutant might have a fatty acid phenotype or *cjd1* mutants might be defective in chloroplast division.

While this hypothesis was partially supported by the initial observation that the original *cjd1-2* mutant line had abnormally large chloroplasts, closer analysis revealed a lack of pleiotropy for *cjd1* and *arc6* mutants. The first line of evidence is that the *cjd1-1* mutant has normal chloroplast size and number (Fig. S4a). Because the abnormal chloroplast trait was observed only for *cjd1-2* we hypothesized it was caused by a second unlinked mutation. To test this idea, *cjd1-2* was backcrossed to isogenic Columbia (Col) wild-type and segregation analysis of the chloroplast morphology phenotype was performed on the F_2_ population. Based upon analysis of 34 F_2_ plants, it is apparent that the mutation causing the chloroplast morphology phenotype is unlinked to the *cjd1*-2 mutation (Table S1). However, to further ensure that the fatty acid phenotype observed for *cjd1-2* was not linked to abnormal chloroplasts, 5 F_2_ plants with normal chloroplasts and also homozygous for the *cjd1-2* insertion were harvested for seed and their progeny analyzed for fatty acid content (Fig. S5). These lines possessed the mutant fatty acid phenotype associated with *cjd1-2*. Further evidence for lack of pleiotropy is that *arc6-5* (Sail_693_G04) has wild-type leaf fatty acid composition despite the abnormal chloroplast number and size (Fig. S4).

Double mutant analysis between genes of related function can reveal novel ‘synthetic’ phenotypes, ranging from enhancement of one or more single mutant phenotypes to novel syndromes including inviability [47]–[49]. To explore the possible relationship between these two J-like domain proteins, *cjd1-1/arc6-5* double mutant was generated by crossing the *arc6-5* mutant allele to *cjd1-1* and genotyping F_2_ progeny. The double mutant possessed chloroplasts identical to *arc6-5* and a fatty acid profile similar to *cjd1-1* fatty acid profile (Fig. S4b,c). Therefore, these genetic analyses failed to establish a functional relationship between ARC6 and CJD1 proteins.

## Discussion

Despite the tremendous amount of information known about plant acyl-lipid metabolism [17], genetics continues to reveal new components that influence leaf fatty acid composition [16] [4], [50]–[52]. In this manuscript we describe an aberrant Arabidopsis fatty acid phenotype caused by mutation of a gene of previously unknown function (At1g08640). The *cjd1-1* mutant was identified in a large-scale reverse genetics screen of T-DNA mutants in nuclear genes encoding chloroplast-targeted proteins (The Chloroplast 2010 Project; http://www.plastid.msu.edu/). The screen was designed with biological and process replication not used in the original forward genetic mutant screens, in hopes of finding mutants with more subtle changes in leaf FAMEs. This was successful since mutations in *CJD1* cause mildly increased accumulation of less highly unsaturated FAMEs (especially 16∶1Δ7 and 18∶1Δ9), while polyunsaturated FAMEs (16∶3 and 18∶3) decrease (Fig. 1c). The changes in fatty acid profiles are more pronounced in the chloroplastic lipids MGDG and DGDG (Table 1), consistent with results indicating that CJD1 protein is located in the chloroplast inner envelope membrane (Fig. 3).

A topological model (Fig. 3d) with CJD1 located in the chloroplast inner envelope membrane is based on various lines of evidence. Fluorescence microscopy of CJD1-GFP fusion lines indicates that CJD1 is plastidic (Fig. 3a). Fractionation of chloroplasts from transgenic CJD1-GFP lines and *in vitro* chloroplast import assays further refined the location of CJD1 to chloroplast membranes. The observation that translocated CJD1 was not accessible to Trypsin degradation (Fig. 3c) suggests that it resides either in the inner envelope or thylakoid membranes. We favor inner envelope localization because of multiple published proteomics studies demonstrating CJD1 protein in Arabidopsis [28]; [30] and pea [29] chloroplast envelope preparations. In addition, Y2H results show that the CJD1 J-like domain has the ability to interact with the N-terminus of the inner envelope protein ARC6, which extends into the stroma from the chloroplast inner envelope (Fig. 3d) [22].

An inconsistency with the topological model shown in Figure 3d is that the CJD1-GFP fusion protein is also insensitive to Trypsin digestion. This is despite the fact that GFP is relatively large (∼28 kD) and predicted in our model to be in the intermembrane space. While there are published cases of Trypsin-insensitive proteins with domains extending into the intermembrane space [23], [53], we cannot rule out the possibility that the CJD1-GFP C-terminus extends into the stroma.

While the pattern of fatty acid changes in *cjd1* mutants is reminiscent of defects in the chloroplast 16∶1/18∶1 desaturase (*FAD6*) [12], [54], the change of magnitude in fatty acid levels in *cjd1* mutants is less pronounced. This suggests that CJD1 may be involved in modulating desaturase activity, either by protein interaction or through a less direct mechanism such as altering desaturase gene expression. A direct test for influence on FAD6 protein accumulation or enzyme activity was not possible due to lack of antibody reagents or *in planta* enzyme activity assays. An alternative approach, Y2H screening did not identify desaturases or other proteins involved in acyl-lipid metabolism as interacting partners (Table 2). Similarly, pull-down experiments with anti-GFP antiserum and the CJD1-GFP transformed lines in Fig. 3a–b failed to reveal interactions with known enzymes of lipid metabolism (I. Ajjawi, unpublished). Finally, no difference in mRNA accumulation was observed for the chloroplast fatty acid desaturases *FAD5*, *6* and *7* between wild type and *cjd1* mutants (Fig. S2). Because these are negative results, it is not possible to assess the influence of CJD1 protein on plastidic desaturase activity.

Several lines of evidence indicate that CJD1 contains a region related to J domains including that found in *E. coli* HSP40 (see Fig. 4a for a schematic comparison of types of proteins with J and J-like domains) [25]. The first 91 N-terminal amino acids of mature CJD1 protein are predicted by homology modeling to fold into 5 helices. This structure is quite similar to the J domain of the DnaJ homologue dnj-2 from *Caenorhabditis elegans*, and helices 1–3 are reminiscent of the *E. coli* J domain structure (Fig. S3). In contrast, despite the predicted similarity in 3-D structure, the CJD1 protein is missing the HPD motif between helices 2 and 3 that is thought to be essential for the interaction of the J domain with HSP70 [55] and is thus a ‘J-like’ domain protein.

Our results are consistent with the hypothesis that CJD1 is not a co-chaperone. First, it is missing the HPD triptych associated with J-domains [25] (Fig. 2). Second, unlike the *E. coli* J-domain, expression of the CJD1_60–164_ domain does not reverse the temperature sensitivity of an *E. coli dnaJ*/*cbpA* double knockout mutant (Fig. 4c). Furthermore, CJD1_(60–164)_ did not give a positive Y2H interaction result when tested with each of two stromal HSP70 proteins, in contrast to positive results with an HSP40 J-domain (Fig. 4b). Finally, Y2H screening of an Arabidopsis cDNA library also failed to identify HSP70s as interactors (Table 2).

In contrast, Y2H library screening for proteins that interact with the CJD1 J-like domain identified multiple clones expressing the stromal region of the chloroplast inner envelope protein ARC6. Mutation of *ARC6* results in a small number of highly enlarged chloroplasts per cell because the protein plays a central role in chloroplast division by coordinating the stromal and outer envelope division components [22], [45]. Both interacting regions included the J-like domain. This is reminiscent of published results showing that the yeast J-like protein TIM16 interacts with the HSP40 protein TIM14 to inhibit the latter protein's co-chaperone activity, which is necessary for protein translocation into the mitochondrion [56]–[59]. The interactions involve helices II and III of the J and J-like domains, but require amino acids outside of the TIM14 J domain for function. This is analogous to our observation that both the ARC6 J-like domain and the adjacent CR are necessary for positive Y2H interaction with CJD1.

The Y2H result led to the hypothesis that ARC6 and CJD1 interaction influences the *in vivo* functions of these proteins. However, mutant analysis failed to reveal evidence for a direct functional relationship between CJD1 and ARC6. Single *arc6-5* mutants have normal fatty acid content and *cjd1* mutations do not influence chloroplast morphology (Fig. S4). Furthermore, *cjd1-1/arc6-5* double mutants fail to show more extreme ‘synthetic’ phenotypes than the single mutants, as is sometimes seen for genes whose products have related functions [47]. Genetic redundancy may account for the lack of appreciable phenotypes. In fact, a total of 89 Arabidopsis J proteins have been identified and catalogued [42] and multiple J-like proteins are also found in the Arabidopsis genome.

In summary, we have demonstrated that mutation of the J-like domain protein CJD1 affects acyl-lipid metabolism in Arabidopsis. The observation of interaction between two J-like domains in the Y2H assay parallels the observation of direct interaction of the J-like domain of yeast Tim16 with the J domain of Tim14 [56]. This result suggests that the repertoire of J and J-like domain protein interactions may be more widespread than currently documented. Whether the CJD1 and ARC6 proteins interact within the chloroplast stroma and the functional significance of such a complex remains to be demonstrated.

## Materials and Methods

### Plant materials, growth conditions, plant genotyping and RT-PCR

All *A. thaliana* lines used in this study are in the Col genetic background. The c*jd1* mutants Salk_032130C (*cjd1-1*) and Salk_039694 (*cjd1-2*) and the *arc6* T-DNA mutant Sail_693_G04 (*arc6-5*) were obtained from the Arabidopsis Biological Resource Center (ABRC). Genotyping of *cjd1-1* and *cjd1-2* was performed as described in [4] and genotyping of *arc6-5* is described in [22]. RT-PCR analysis of *cjd1* mutants to check for *CJD1*, *FAD5*, *FAD6* and *FAD7* transcript levels was conducted as previously described [4]. The following are sequences for the primer sets used in the RT-PCR reactions: *CJD1*, 5′-atggctcccgcactatctac-3′ and 5′-ttatctgtaaaacgacgcta-3′; *FAD5*, 5′-tagtttggtgggagagagaa-3′ and 5′-gaaccaaaacccatcaagtg-3′; *FAD6*, 5′-acagggaacagttagcagaa-3′ and 5′-taacatgttggttttggcgt-3′; *FAD7*, 5′-acgtcgctatcgtctttgca-3′ and 5′-tgcagtccaacaagcagtag-3′. Growth conditions for plants grown in the Chloroplast 2010 pipeline were described in detail by [5], unless otherwise indicated.

### Lipid and fatty acid analysis

Total leaf FAMEs were analyzed as described in [5]. Leaf glycerolipids were extracted and subject to thin-layer chromatography as previously described [36] on activated ammonium sulfate-impregnated silica gel TLC plates (Si250PA; Mallinckrodt, Baker, NJ, USA) using a solvent system of acetone/toluene/water (91/30/7.5, v∶v∶v). Lipids were stained by exposure to iodine vapor for 30 seconds and silica material containing MGDG, DGDG, PE, PC and PG was scraped with a razor blade into a glass reaction tube. FAMEs from these fractions were prepared as previously described [16].

### Generation of a CJD1-GFP fusion construct and plant transformation

Forward primer 5′-accatggctcccgcactatctac-3′ and reverse primer 5′-ttctgtaaaacgacgctataa-3′ were used to amplify the *CJD1* open reading frame (gene model At1g08640.1) without the stop codon from wild-type Col cDNA. The resulting *CJD1* PCR fragment was cloned into pCR®8/GW/TOPO® (Invitrogen, Carlsbad, CA, USA) and the C-terminus of *CJD1* was fused to the N-terminus of *GFP* by LR cloning (Invitrogen) into the Gateway-compatible plant expression vector pMDC85 [60]. The resulting binary vector, pMDC85-CJD1-GFP was transformed into *Agrobacterium tumefaciens* strain GV3101 and in turn, used to transform wild-type Col plants by floral dip [61]. Transgenic lines were screened for hygromycin B (25 mg mL^−1^) resistance and those lines that exhibited segregation ratios consistent with the presence of a single transgene locus were used for GFP visualization and immunoblot analysis. These plants were grown at 21°C for a 16-h light/8-h dark photoperiod and exposed to an irradiance of 100 µmol m^−2^ s^−1^.

### Immunoblotting and GFP visualization

CJD1-GFP fluorescence was directly examined by confocal microscopy as described previously [16]. For immunoblotting, chloroplasts were isolated from approximately 6 g of leaf tissue obtained from 2-week-old Arabidopsis seedlings essentially as previously described [62]; however, intact chloroplasts were recovered using a modified 30% Percoll cushion rather than a linear Percoll gradient. Recovered intact chloroplasts were assayed for protein content using the Bradford assay (BioRad, Hercules, CA, USA). An equal amount of total protein obtained either from untransformed plants or transgenic lines expressing CJD1-GFP was subsequently used for fractionation analysis. Briefly, intact chloroplasts were pelleted and then resuspended in lysis buffer (25 mM Hepes pH 8.0, 4 mM MgCl_2_), and incubated on ice for 20 minutes. Lysed chloroplasts were centrifuged and crude membrane and supernatant fractions were recovered. All supernatant fractions were acetone precipitated for 30 minutes on ice, centrifuged, and the resultant pellets were solubilized in 2× SDS electrophoresis sample buffer. Likewise, all membrane pellet fractions were directly solubilized in 2× sample buffer. Ten uL of each sample was resolved by SDS-PAGE and further analyzed by Western blotting [63] using SuperSignal® West Pico (Thermo Scientific, Rockford, IL, USA) as the chemiluminescence detection system. Anti-GFP (ab290) was purchased from Abcam Inc. (Cambridge, MA, USA) and anti-HSP70 [29] and anti-HSP93 [30] were used as controls.

### Chloroplast import experiments

The cDNA encoding *CJD1* was amplified from wild-type Col cDNA using forward primer 5′-atggctcccgcactatctac-3′ and reverse primer 5′-ttatctgtaaaacgacgcta-3′. The resulting PCR product was cloned into pCR®2.1-TOPO (Invitrogen) following the manufacturer's instructions. To transfer the CJD1-GFP fusion protein into a vector suitable for *in vitro* translations, the CJD1-GFP cassette was PCR amplified from pMDC85-CJD1-GFP using primers 5′-atggctcccgcactatctac-3′ and 5′-cttagtggtggtggtggtgg-3′ and cloned into pCR®8/GW/TOPO®. The *RBCS* [64] and *ARC6* [23] genes were used as controls. Precursor proteins were radiolabeled using [35S]-methionine and translated with TNT® Coupled Reticulocyte Lysate System (Promega Corporation, Madison, WI, USA) according to the manufacturer's protocol. Intact chloroplasts were isolated from 8- to 12-day-old pea seedlings and purified over a Percoll gradient as previously described [65]. Intact pea chloroplasts were reisolated and resuspended in import buffer (330 mM sorbitol, 50 mM Hepes-KOH, pH 8.0) at a concentration of 1 mg chlorophyll/mL. Import assays were performed as described in [65] and Trypsin sensitivity assays were performed as described by [23].

### *E. coli* temperature sensitivity experiments

The coding sequence for CJD1 amino acids 60 to 164 was amplified using primers 5′-atggcttcgtctgcggctggtaatccaca-3′ and 5′-ctcgagattcttggaggacctggaaa-3′, cloned into pCR®2.1-TOPO and transferred into the *EcoR*I site of pBAD18 [66] resulting in pBAD18(CJD1_60–164_). The *dnaJ*/*cbpA E. coli* temperature sensitive double mutant (WKG90) and the pBAD plasmids carrying either *E. coli* DnaJ (pWKG90) or the J domain of *E.coli* DnaJ (pWKG100) were all a kind gift from Dr. William Kelley [39]. After transformation into the *E.coli dnaJ*/*cbpA* double mutant strain, the strains were spotted at different dilutions onto LB ampicillin (20 µg/ml) plates supplemented with either 0.01%, 0.1%, 0.5%, or 1%, w/v arabinose, or no arabinose, and allowed to grow at 37°C, 39°C, 40.5°C and 42°C. Results for cells grown at 39°C and 42°C on plates supplemented with 0.5% w/v arabinose are shown in Fig. 4.

### Bioinformatics

CJD1 homologues were identified by BLAST search [27]. The protein sequence available at GenBank (GenBank protein ID, 159491044) for the *C. reinhardtii* homologue was fused to a glycosyl hydrolase and was therefore manually trimmed to exclude the glycosyl hydrolase. The sequences for *G. max* gene Glyma03g41110 and *Z. mays* gene GRMZM2G050118 had not been deposited at GenBank and were obtained from Phytozyme (http://www.phytozome.net/). The multiple sequence alignment shown in Fig. 2 was computed by ClustalW (1.83) [67] and shading of the conserved amino acid residues was done using the BOXSHADE tool (3.21) both available at the Swiss EMBnet node server (http://www.ch.embnet.org/index.html). Phylogenetic analysis was performed as previously described [16]. Secondary structure profiling and homology modeling were performed using tools available on the BioInfoBank MetaServer (http://meta.bioinfo.pl/submit_wizard.pl) [37].

### Yeast 2-hybrid analysis

Yeast 2-hybrid screening was performed by Hybrigenics (Paris, France). The DNA coding sequence for CJD1 amino acids 60 to 164 was PCR-amplified and cloned into pB27 as a C-terminal fusion to LexA (N-LexA-At1g08640-C). The construct was checked by sequencing the entire insert and used as a bait to screen a random-primed *A. thaliana* one-week old seedling cDNA library constructed into pP6. pB27 and pP6 derive from the original pBTM116 [68] and pGADGH plasmids, respectively. 98.8 million clones (10-fold the complexity of the library) were screened using a mating approach with Y187 (*MAT*α) and L40ΔGal4 (*MAT*a) yeast strains as previously described [69]. 16 His+ colonies were selected on a medium lacking tryptophan, leucine and histidine. The prey fragments of the positive clones were amplified by PCR and sequenced at their 5′ and 3′ junctions. The resulting sequences were used to identify the corresponding interacting proteins in GenBank.

Directed Y2H experiments were conducted using Clontech's (Mountain View, CA, USA) MatchMaker GAL4 Two-Hybrid System 3 kit following the manufacturer's instructions. The coding sequence for CJD1 amino acids 60–164 was excised from pBAD18(CJD1_60–164_) and cloned into pGADT7 using *EcoR*I sites. ARC6_154–331_ was amplified by PCR with primers 5′-ttttttcatatgcttgatgatgaagaagctacag-3′ and 5′-ttttttcccgggttactgctcagcagctgtcattcgtaa-3′, using the full-length ARC6 cDNA clone U19395 (ABRC) as a template. The PCR product was cloned into pGBKT7 vector using *Nde*I and *Xma*I. pGBK-ARC6_84–331_, pGBK-ARC6_84–169_, pGBK-cpHSP70-1, and pGBK-cpHSP70-2 constructs were made using the Gateway System (Invitrogen). ARC6_84–331_ and ARC6_84–169_ were PCR amplified using primer sets 5′-ggggacaagtttgtacaaaaaagcaggcttcatggtccccatccccattgatttc-3′ and 5′-ggggaccactttgtacaagaaagctgggtcctactgctcagcagctgtcattcgta-3′; 5′- ggggacaagtttgtacaaaaaagcaggcttcatggtccccatccccattgatttc-3′ and 5′-ggggaccactttgtacaagaaagctgggtcctacttatcccaaggaacatcagtga-3′, respectively. Mature cpHSP70-1 (aa77–718), was amplified from a full-length cDNA clone (pGEMT-cpHsc70-1, a gift from Hsou-min Li lab) using primers 5′-ggggacaagtttgtacaaaaaagcaggcttcatgaacgagaaggttgttggaattgat-3′ and 5′-ggggaccactttgtacaagaaagctgggtctcattggctgtctgtgaagtcag-3′. Mature cpHSP70-2 (aa77–718), was amplified from full-length cDNA clone C105236 (ABRC) using primers 5′-ggggacaagtttgtacaaaaaagcaggcttcatgaacgagaaagtcgtcggaatc-3′ and 5′-ggggaccactttgtacaagaaagctgggtcttaattgctgtctgtgaagtca-3′. All PCR products were cloned into pDNOR207 via BP reactions (Invitrogen). Destination constructs were subsequently generated by LR reactions of the respective entry clones with the Gateway destination vector pGBK-GW and pGAD-GW (converted from pGBKT7 and pGADT7, respectively). pGBK-ARC6_84–210_, pGBK-ARC6_84–250_, pGBK-ARC6_84–290_, pGBK-ARC6_120–331_ and pGBK-atDjA24 were also constructed by Gateway cloning, but were subcloned first into pCR®8/GW/TOPO® (Invitrogen). The forward primer 5′-gtccccatccccattgatttc-3′ was used to generate the PCR fragments for ARC6_84–210_, ARC6_84–250_ and ARC6_84–290_, in combination with reverse primers 5′-ccataactaaaaccacatcttg-3′, 5′-ggctacttgctccttcctcctg-3′ and 5′-taccatttagtcttttcgcagc-3′, respectively. The primer pair 5′-ggtttcagcgacgacgcttta-3′ and 5′-gctcagcagctgtcattcgta-3′ was used to amplify ARC6_120–331_, while primer pair 5′-tctcaccaagacaattgcatcagc-3′ and 5′-caccatcgatagctctgctccttgaacc-3′ was used to amplify the J domain (amino acids 32–200) of atDjA24.

## Supporting Information

Figure S1 **Rooted tree indicating the relatedness of predicted CJD1 protein homologues in representative organisms.** The *E. coli* DnaJ protein was used as an outgroup. Boot strapping values >900 are marked by a plus sign, those between 500 and 900 are marked with an open circle and those under 500 by a cross. Protein sequences in addition to those already described in Figure 2 (GenBank Protein ID): E.coli, *Escherichia coli*, 16128009; Micromonas, *Micromonas pusilla* (RCC299), 255073349; Physco, *Physcomitrella patens*, 162675779; Oryza2, *Oryza sativa*,113622873; Populus2, *Populus trichocarpa*, 224123536; Sorghum, *Sorghum bicolor*, 242080423; Ricinus, *Ricinus communis*, 255561927; Medicago, *Medicago truncatula*, 217075548; GlycineM1, *Glycine max*, 255638094; Prochloro2, *Prochlorococcus marinus* (MIT9515), 123200338; Microcoleu, *Microcoleus chthonoplastes* (PCC7420), 254411043; Nodularia, *Nodularia spumigena* (CCY9414), 119513416; NostocP, *Nostoc punctiforme* (PCC7312), 186684227. Locus identifiers for ZeaMays2 and GlycineM2 are GRMZM2G050118 and Glyma03g41110, respectively.
(PDF)

Figure S2 **Steady-state transcript abundance of** ***FAD5*** **,** ***FAD6*** **,** ***FAD7*** **and** ***EF1α*** **in wild-type (WT),** ***cjd1-1*** **and** ***cjd1-2*** **plants.** A, 25 and B, 30 cycles into the RT-PCR analysis. Three biological replicates (1,2,3) were tested.
(PDF)

Figure S3 **CJD1_(60–151)_ homology model.** The model is based on an alignment between CJD1_(60–151)_ and the J domain of the *C. elegans* DnaJ homologue, dnj-2. PyMol software was utilized to create the images. N-termini are displayed in blue while C-termini are colored red.
(PDF)

Figure S4 **Phenotypes of** ***cjd1-1 and arc6-5*** **single and double mutants.** A, Leaf petiole cell images. B, and C, Leaf methyl esters of these lines determined by GC-FID (n = 4 for WT, *arc6-5* and *cjd1-1*, n = 3 for *arc6-5/cjd1-1*). Statistically significant differences relative to wild type (Student's *t* test *P*<0.01) are indicated with asterisks.
(PPT)

Figure S5 **Leaf fatty acid composition of** ***cjd1-2*** **backcrossed to wild type.** A, and B, fatty acid composition (mol%) of five independent F3 progeny of *cjd1-2* backcrossed to wild-type Col (WT). The error bars represent the standard deviation of four biological replicates. Statistically significant differences relative to WT (Student's t test P<0.01) are indicted with asterisks.
(PPT)

Table S1 **Segregation analysis of the chloroplast morphology phenotype found in** ***cjd1-2*** **.** Homo/Het, homozygous or heterozygous for *cjd1-2* T-DNA, respectively. WT, *cjd1-2* T-DNA was not detected by PCR. +/− Presence or absence of abnormal chloroplasts, respectively.
(PDF)
