# Supplementary figures and images for: A J-Like Protein Influences Fatty Acid Composition of Chloroplast Lipids in Arabidopsis
Source: PLoS One. 2011 Oct 18;6(10):e25368. doi: 10.1371/journal.pone.0025368 (PMC3196505; doi:10.1371/journal.pone.0025368)

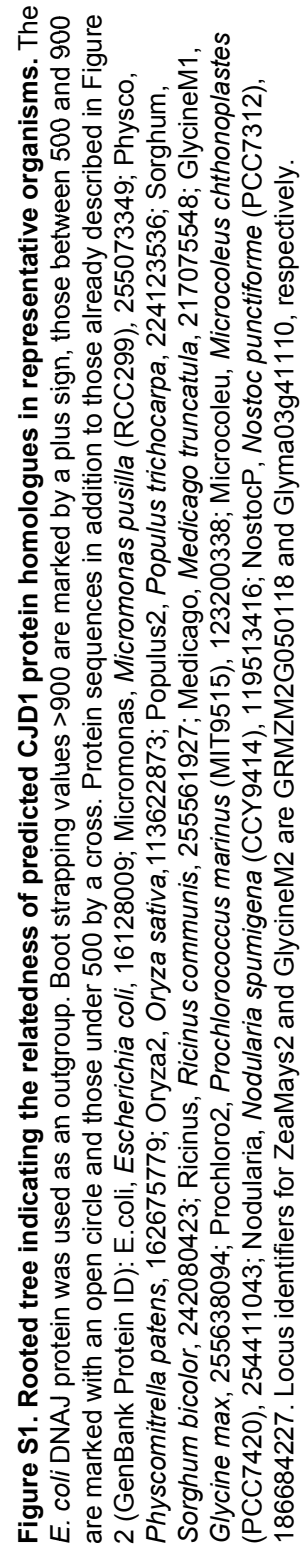

Supplement: Figure S1 — Rooted tree indicating the relatedness of predicted CJD1 protein homologues in representative organisms. The E. coli DnaJ protein was used as an outgroup. Boot strapping values >900 are marked by a plus sign, those between 500 and 900 are marked with an open circle and those under 500 by a cross. Protein sequences in addition to those already described in Figure 2 (GenBank Protein ID): E.coli, Escherichia coli, 16128009; Micromonas, Micromonas pusilla (RCC299), 255073349; Physco, Physcomitrella patens, 162675779; Oryza2, Oryza sativa,113622873; Populus2, Populus trichocarpa, 224123536; Sorghum, Sorghum bicolor, 242080423; Ricinus, Ricinus communis, 255561927; Medicago, Medicago truncatula, 217075548; GlycineM1, Glycine max, 255638094; Prochloro2, Prochlorococcus marinus (MIT9515), 123200338; Microcoleu, Microcoleus chthonoplastes (PCC7420), 254411043; Nodularia, Nodularia spumigena (CCY9414), 119513416; NostocP, Nostoc punctiforme (PCC7312), 186684227. Locus identifiers for ZeaMays2 and GlycineM2 are GRMZM2G050118 and Glyma03g41110, respectively. (PDF) [file pone.0025368.s001.pdf]
