# Supplementary material for: A J-Like Protein Influences Fatty Acid Composition of Chloroplast Lipids in Arabidopsis
Source: PLoS One. 2011 Oct 18;6(10):e25368. doi: 10.1371/journal.pone.0025368 (PMC3196505; doi:10.1371/journal.pone.0025368)
Supplement: Figure S2 — Steady-state transcript abundance of FAD5 , FAD6 , FAD7 and EF1α in wild-type (WT), cjd1-1 and cjd1-2 plants. A, 25 and B, 30 cycles into the RT-PCR analysis. Three biological replicates (1,2,3) were tested. (PDF) [file pone.0025368.s002.pdf]

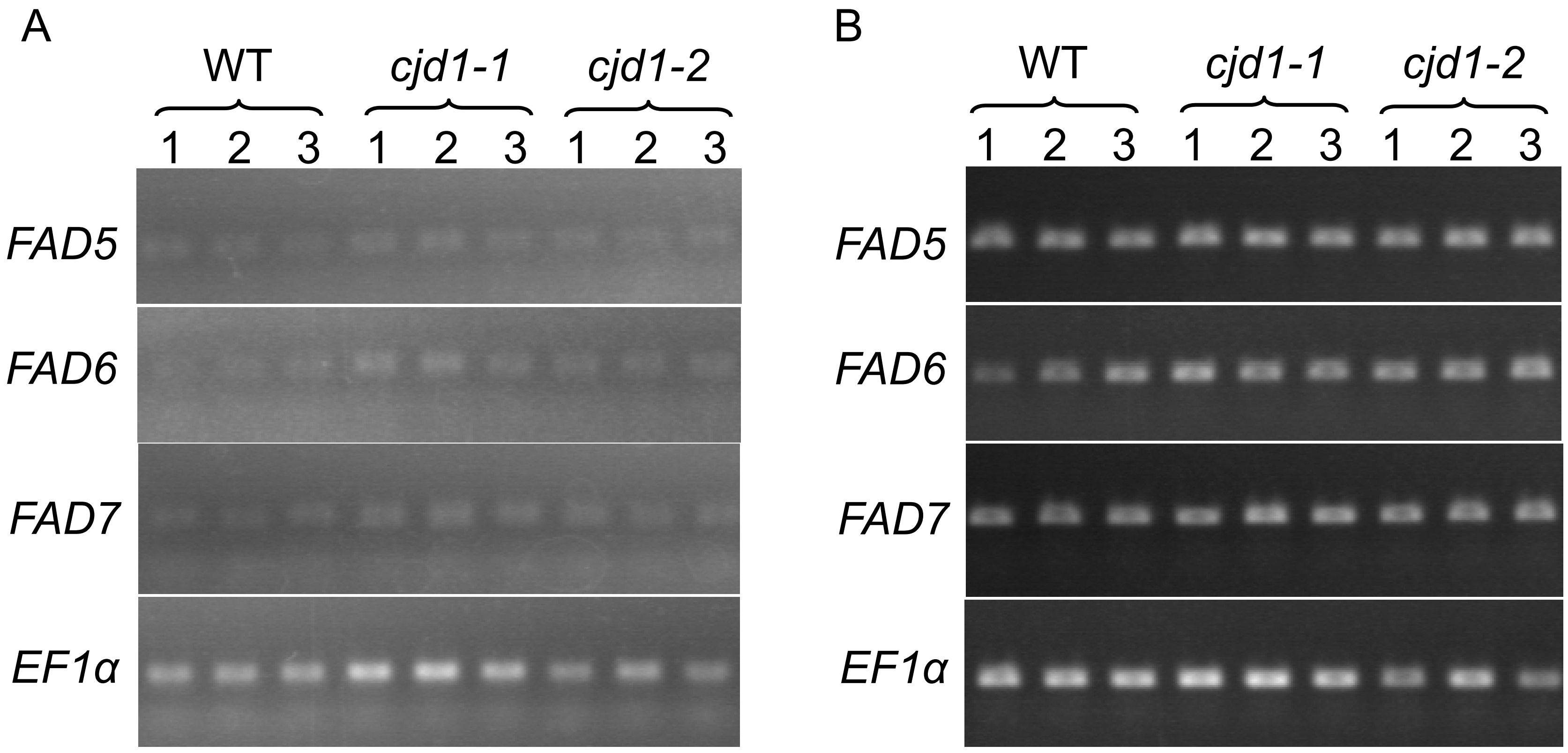

Figure S2. Steady-state transcript abundance of *FAD5*, *FAD6*, *FAD7* and *EF1α* in wild-type (WT), *cjd1-1* and *cjd1-2* plants. A, 25 and B, 30 cycles into the RT-PCR analysis. Three biological replicates (1,2,3) were tested.
