# Supplementary material for: A J-Like Protein Influences Fatty Acid Composition of Chloroplast Lipids in Arabidopsis
Source: PLoS One. 2011 Oct 18;6(10):e25368. doi: 10.1371/journal.pone.0025368 (PMC3196505; doi:10.1371/journal.pone.0025368)
Supplement: Figure S3 — CJD1(60–151) homology model. The model is based on an alignment between CJD1(60–151) and the J domain of the C. elegans DnaJ homologue, dnj-2. PyMol software was utilized to create the images. N-termini are displayed in blue while C-termini are colored red. (PDF) [file pone.0025368.s003.pdf]

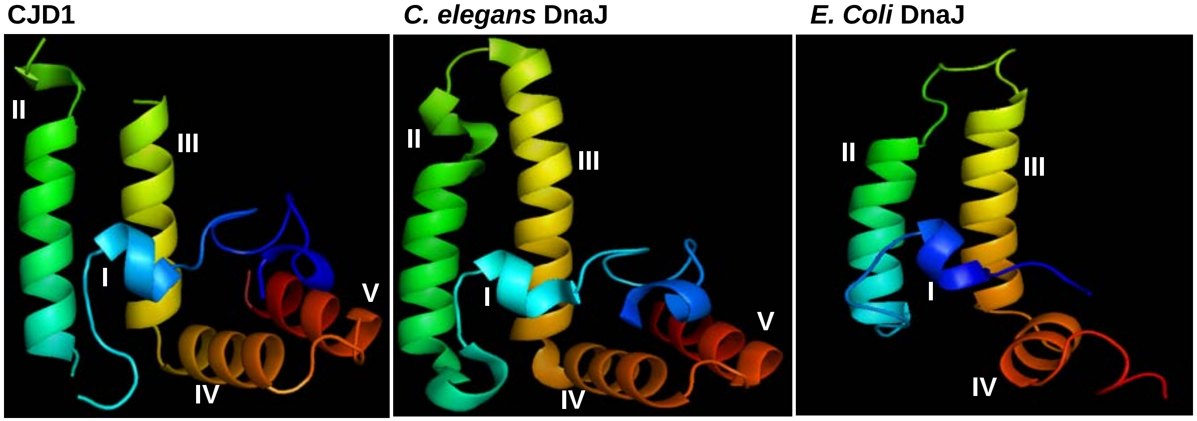

Figure S3. CJD1$_{(60\ -151)}$ homology model. The model is based on an alignment between CJD1$_{(60\ -151)}$ and the J domain of the *C. elegans* DnaJ homologue, dnj-2. PyMol software was utilized to create the images. N-termini are displayed in blue while C-termini are colored red.
