# Supplementary material for: A J-Like Protein Influences Fatty Acid Composition of Chloroplast Lipids in Arabidopsis
Source: PLoS One. 2011 Oct 18;6(10):e25368. doi: 10.1371/journal.pone.0025368 (PMC3196505; doi:10.1371/journal.pone.0025368)
Supplement: Figure S4 — Phenotypes of cjd1-1 and arc6-5 single and double mutants. A, Leaf petiole cell images. B, and C, Leaf methyl esters of these lines determined by GC-FID (n = 4 for WT, arc6-5 and cjd1-1, n = 3 for arc6-5/cjd1-1). Statistically significant differences relative to wild type (Student's t test P<0.01) are indicated with asterisks. (PPT) [file pone.0025368.s004.ppt]

## Slide 1
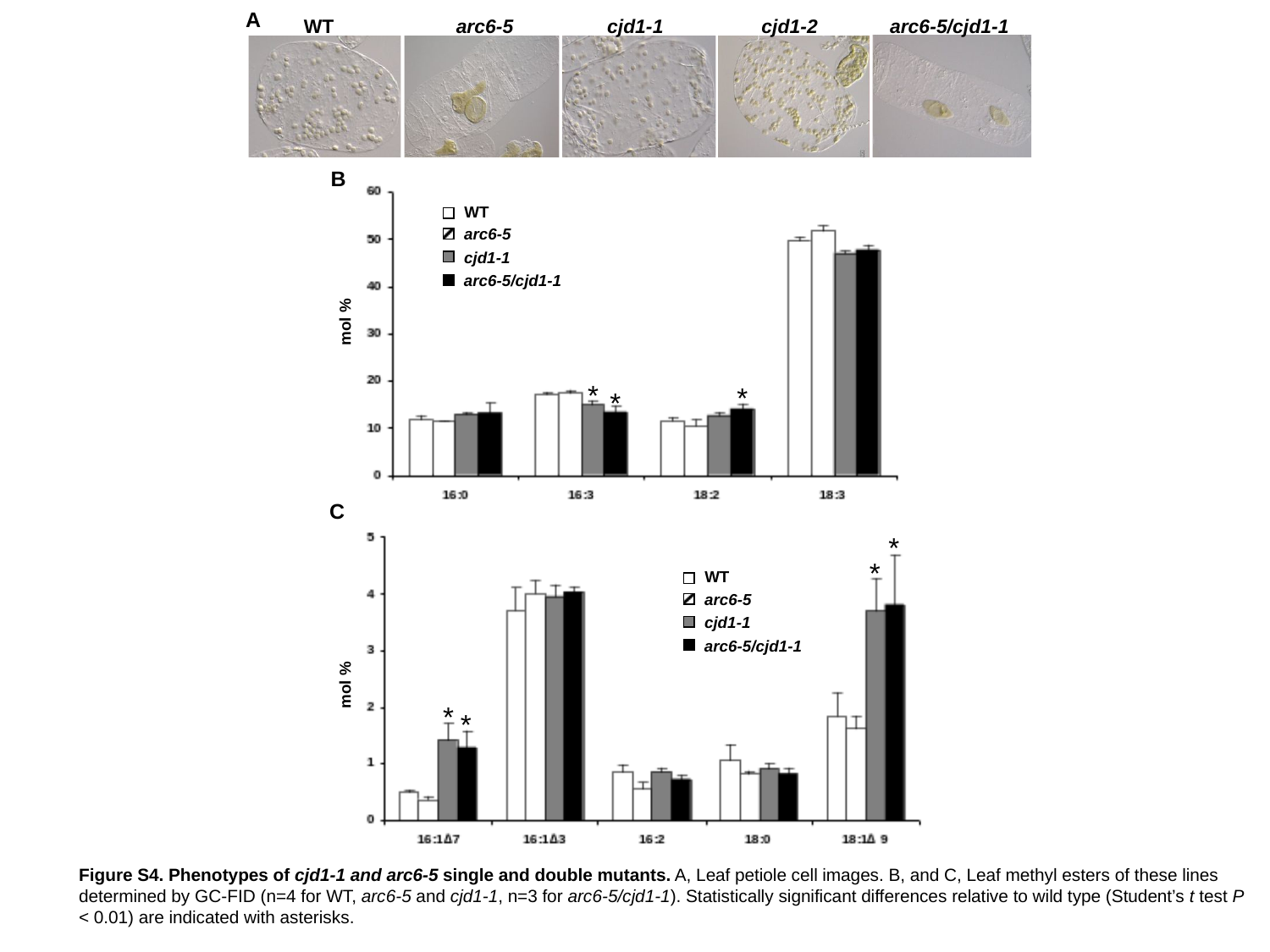

A
WT
arc6-5
cjd1-1
cjd1-2
arc6-5/cjd1-1
B
WT
arc6-5
cjd1-1
arc6-5/cjd1-1
mol %
*
*
*
C
*
*
WT
arc6-5
cjd1-1
arc6-5/cjd1-1
mol %
*
*
Figure S4. Phenotypes of cjd1-1 and arc6-5 single and double mutants. A, Leaf petiole cell images. B, and C, Leaf methyl esters of these lines determined by GC-FID (n=4 for WT, arc6-5 and cjd1-1, n=3 for arc6-5/cjd1-1). Statistically significant differences relative to wild type (Student’s t test P < 0.01) are indicated with asterisks.
