# Supplementary material for: A J-Like Protein Influences Fatty Acid Composition of Chloroplast Lipids in Arabidopsis
Source: PLoS One. 2011 Oct 18;6(10):e25368. doi: 10.1371/journal.pone.0025368 (PMC3196505; doi:10.1371/journal.pone.0025368)
Supplement: Figure S5 — Leaf fatty acid composition of cjd1-2 backcrossed to wild type. A, and B, fatty acid composition (mol%) of five independent F3 progeny of cjd1-2 backcrossed to wild-type Col (WT). The error bars represent the standard deviation of four biological replicates. Statistically significant differences relative to WT (Student's t test P<0.01) are indicted with asterisks. (PPT) [file pone.0025368.s005.ppt]

## Slide 1
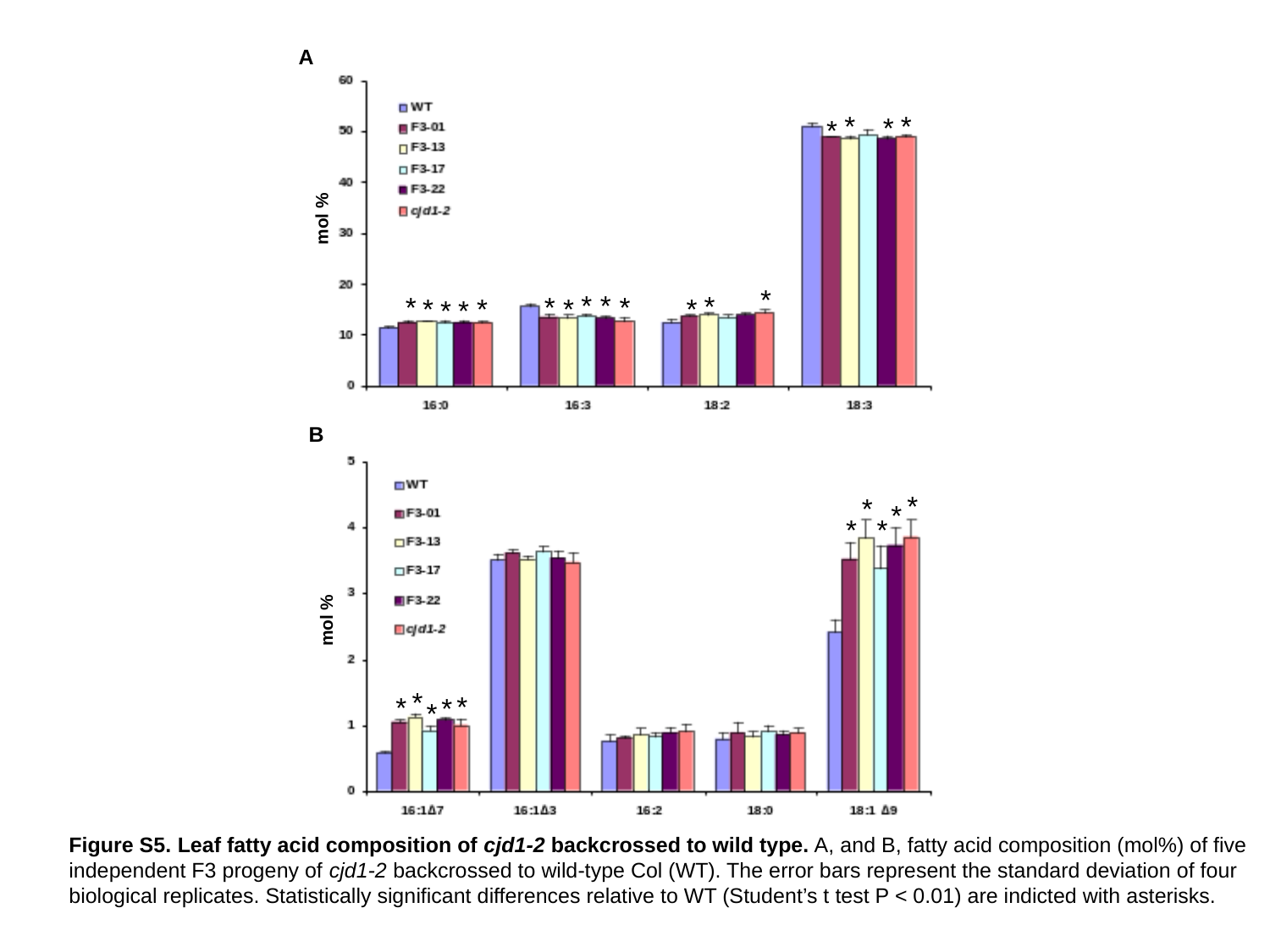

A
*
*
*
*
mol %
*
*
*
*
*
*
*
*
*
*
*
*
*
B
*
*
*
*
*
mol %
*
*
*
*
*
Figure S5. Leaf fatty acid composition of cjd1-2 backcrossed to wild type. A, and B, fatty acid composition (mol%) of five independent F3 progeny of cjd1-2 backcrossed to wild-type Col (WT). The error bars represent the standard deviation of four biological replicates. Statistically significant differences relative to WT (Student’s t test P < 0.01) are indicted with asterisks.
