# Supplementary material for: A J-Like Protein Influences Fatty Acid Composition of Chloroplast Lipids in Arabidopsis
Source: PLoS One. 2011 Oct 18;6(10):e25368. doi: 10.1371/journal.pone.0025368 (PMC3196505; doi:10.1371/journal.pone.0025368)
Supplement: Table S1 — Segregation analysis of the chloroplast morphology phenotype found in cjd1-2 . Homo/Het, homozygous or heterozygous for cjd1-2 T-DNA, respectively. WT, cjd1-2 T-DNA was not detected by PCR. +/− Presence or absence of abnormal chloroplasts, respectively. (PDF) [file pone.0025368.s006.pdf]

Table S1
Segregation analysis of the chloroplast morphology phenotype found in *cjd1-2*

Plant	Genotype	Chloroplast Morphology
1	Homo	-
2	WT	+
3	Homo	-
4	Het	-
5	Homo	-
6	WT	+
7	WT	+
8	WT	-
9	Homo	-
10	Het	-
11	Homo	+
12	WT	-
13	Homo	-
14	WT	-
15	Het	-
16	Het	-
17	Homo	-
18	WT	-
19	WT	+
20	WT	-
21	Het	-
22	Homo	-
23	Het	+
24	WT	-
25	Het	-
26	Het	-
27	Het	-
28	Het	-
29	Homo	+
30	WT	-
31	Het	-
32	Het	-
33	Het	+
34	Het	+

Homo/Het, homozygous or heterozygous for *cjd1-2* T-DNA, respectively. WT, *cjd1-2* T-DNA was not detected by PCR. +/- Presence or absence of abnormal chloroplasts, respectively.
